# Supplementary figures and images for: Structure of the Calvin-Benson-Bassham sedoheptulose-1,7-bisphosphatase from the model microalga Chlamydomonas reinhardtii
Source: eLife. 2025 Apr 1;12:RP87196. doi: 10.7554/eLife.87196 (PMC11961124; doi:10.7554/eLife.87196)

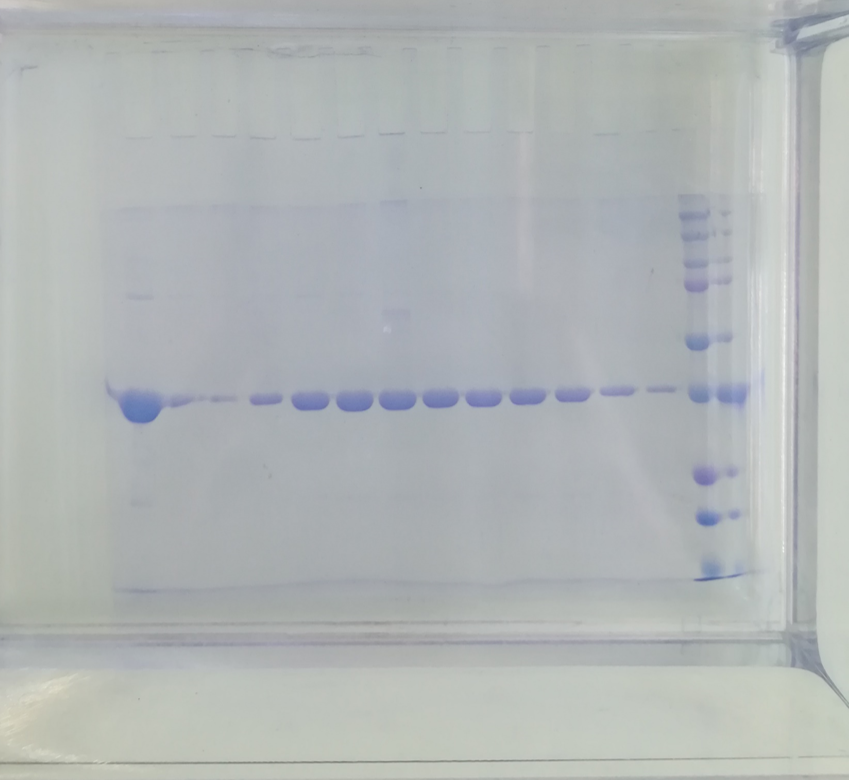

Supplement: Figure 1—figure supplement 1—source data 1. [file elife-87196-fig1-figsupp1-data1.tif]

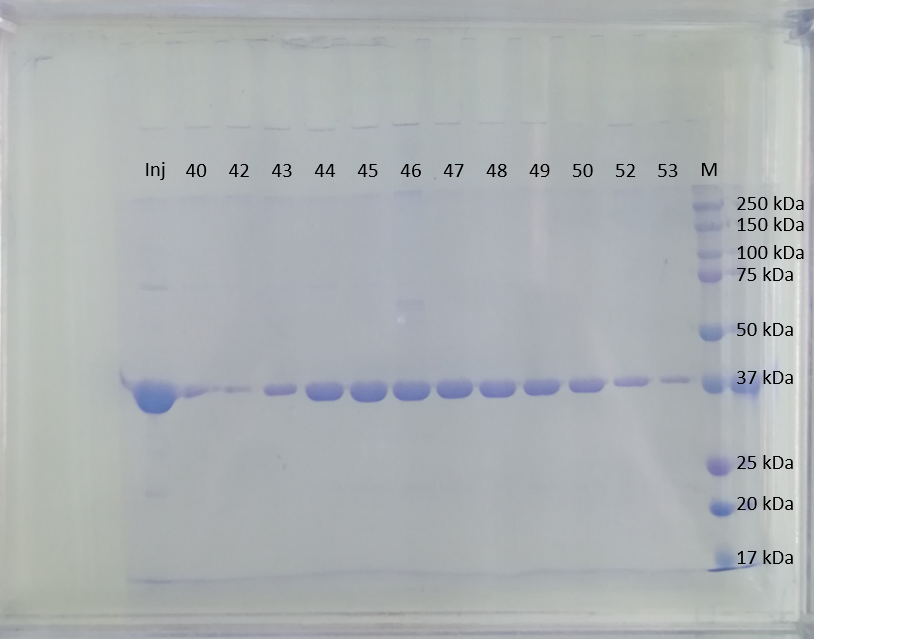

Supplement: Figure 1—figure supplement 1—source data 2. [file elife-87196-fig1-figsupp1-data2.tif]

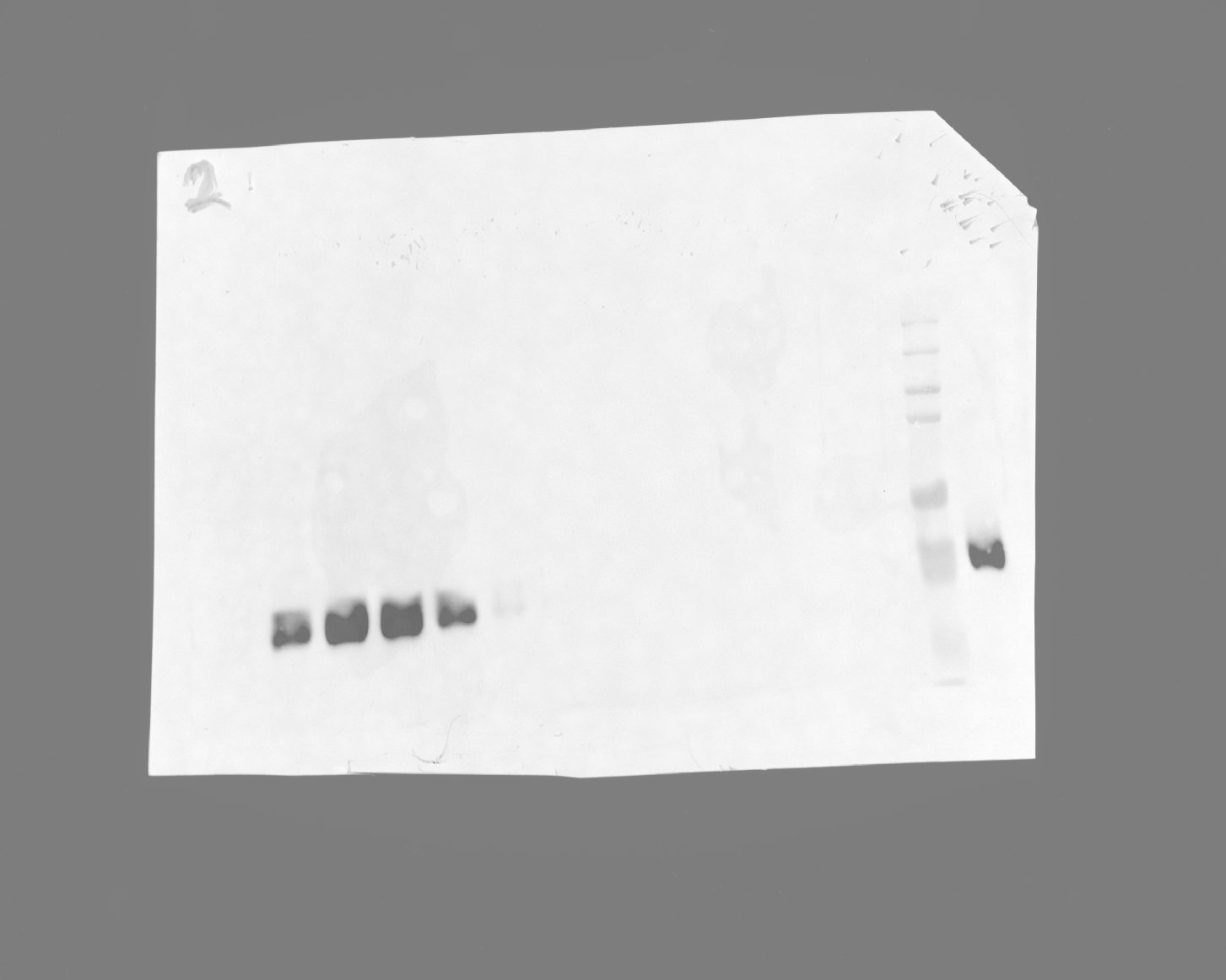

Supplement: Figure 6—source data 1. [file elife-87196-fig6-data1.zip › Figure6-SourceData1/wb-2-uncropped.tif]

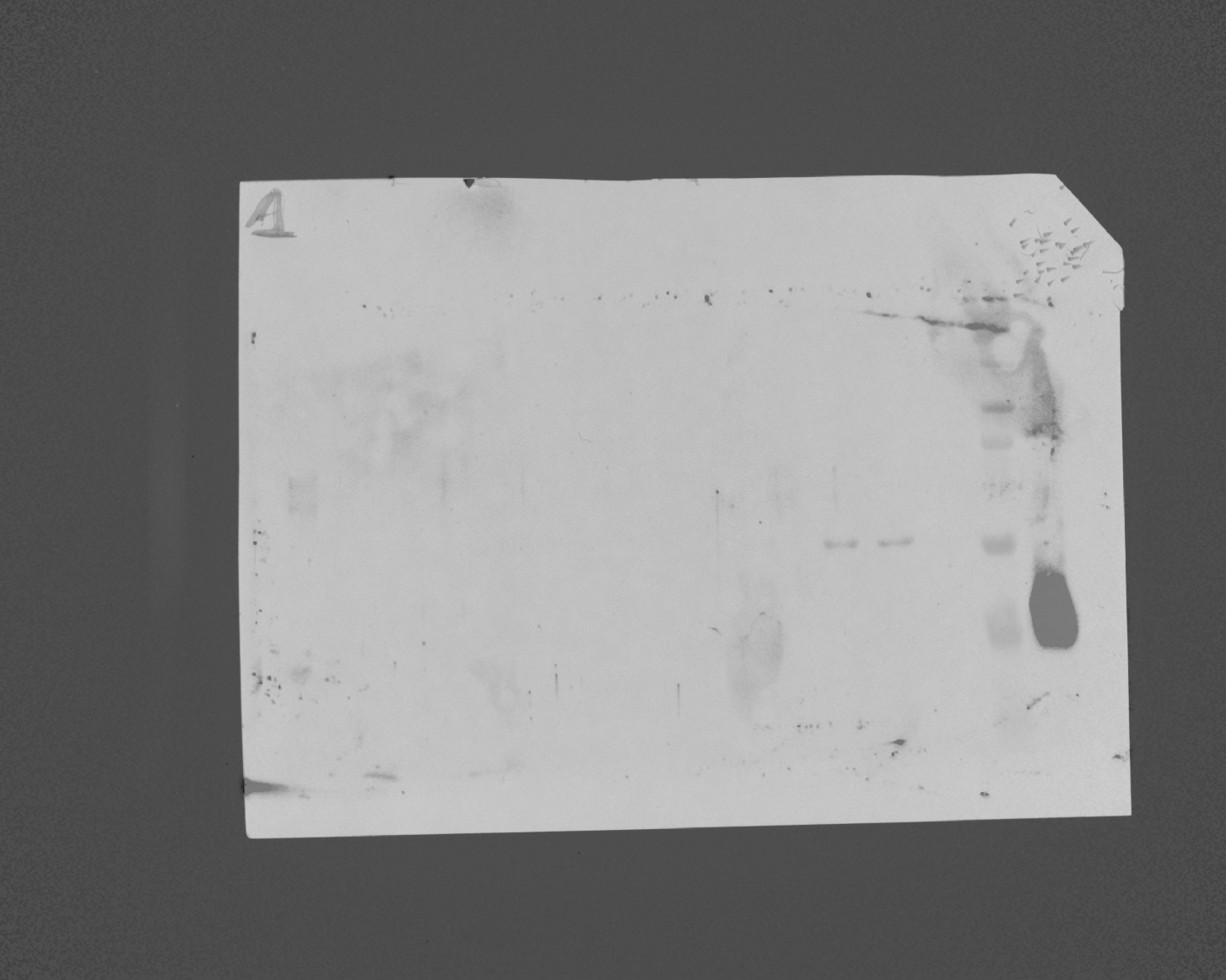

Supplement: Figure 6—source data 1. [file elife-87196-fig6-data1.zip › Figure6-SourceData1/wb-1-uncropped.tif]

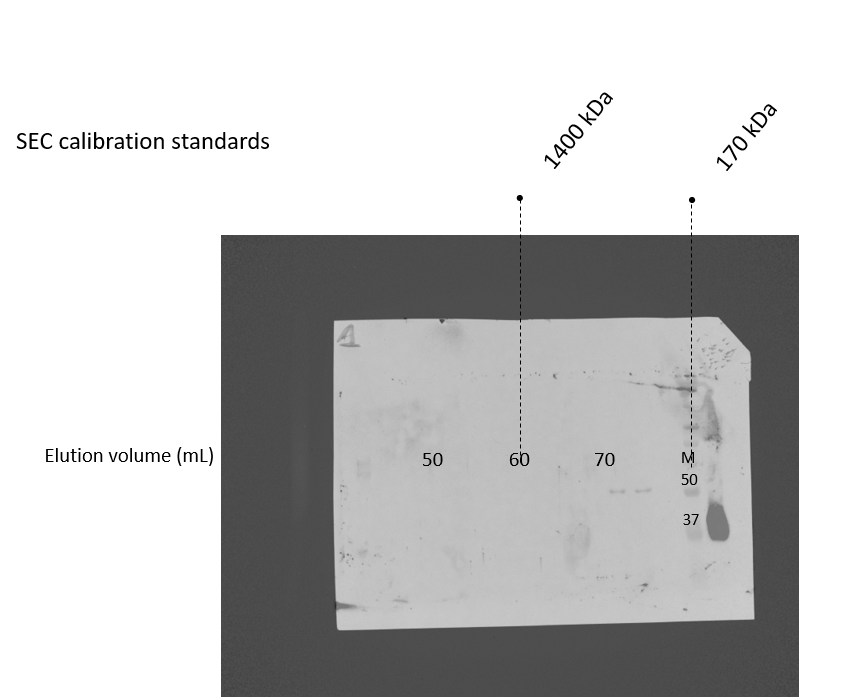

Supplement: Figure 6—source data 2. [file elife-87196-fig6-data2.zip › Figure6-SourceData2/wb-1-uncropped-annotated.tif]

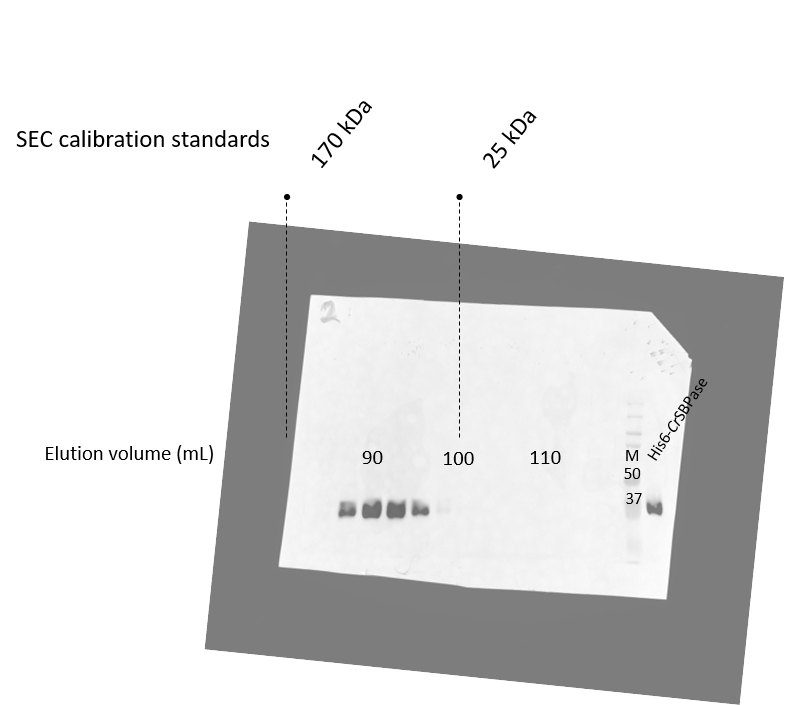

Supplement: Figure 6—source data 2. [file elife-87196-fig6-data2.zip › Figure6-SourceData2/wb-2-uncropped-annotated.tif]
